# Cognitive Training with Neurofeedback Using NIRS Improved Cognitive Functions in Young Adults: Evidence from a Randomized Controlled Trial

**DOI:** 10.3390/brainsci12010005

**Published:** 2021-12-21

**Authors:** Rui Nouchi, Haruka Nouchi, Jerome Dinet, Ryuta Kawashima

**Affiliations:** 1 Department of Cognitive Health Science, Institute of Development, Aging and Cancer (IDAC), Tohoku University, Sendai 980-8575, Japan; haruka.nouchi.e8@tohoku.ac.jp; 2 Smart Aging Research Center (S.A.R.C.), Tohoku University, Seiryo-Machi 4-1, Sendai 980-8575, Japan; ryuta@tohoku.ac.jp; 3 Department of Psychology, Université de Lorraine, F-54000 Nancy, France; jerome.dinet@univ-lorraine.fr; 4 Lorraine Research Laboratory in Computer Science and Its Applications (LORIA), Université de Lorraine, CNRS, INRIA, F-54000 Nancy, France; 5 Department of Functional Brain Imaging, Institute of Development, Aging and Cancer (IDAC), Tohoku University, Sendai 980-8575, Japan

**Keywords:** neurofeedback, cognitive training, cognitive improvement, NIRS, dorsolateral prefrontal cortex

## Abstract

(1) Background: A previous study has shown that cognitive training with neurofeedback (CT-NF) using down-regulation improves cognitive functions in young adults. Neurofeedback has two strategies for manipulating brain activity (down-regulation and upregulation). However, the benefit of CT-NF with the upregulation of cognitive functions is still unknown. In this study, we investigated whether the upregulation of CT-NF improves a wide range of cognitive functions compared to cognitive training alone. (2) Methods: In this double-blinded randomized control trial (RCT), 60 young adults were randomly assigned to one of three groups: CT-NF group, CT alone group, and an active control (ACT) group who played a puzzle game. Participants in the three groups used the same device (tablet PC and 2ch NIRS (near-infrared spectroscopy)) and performed the training game for 20 min every day for four weeks. We measured brain activity during training in all groups, but only CT-NFs received NF. We also measured a wide range of cognitive functions before and after the intervention period. (3) Results: The CT-NF groups showed superior beneficial effects on episodic memory, working memory, and attention compared to the CT alone and ACT groups. In addition, the CT-NF group showed an increase in brain activity during CT, which was associated with improvements in cognitive function. (4) Discussion: This study first demonstrated that the CT-NF using the upregulation strategy has beneficial effects on cognitive functions compared to the CT alone. Our results suggest that greater brain activities during CT would enhance a benefit from CT.

## 1. Introduction

Cognitive training (CT) is a structured and repeated practice to improve specific cognitive functions. Several types of CT have been proposed. CT typically uses cognitive tasks such as working memory [1] and processing speed [2] or brain training games that include key cognitive processes [3,4]. CT has beneficial effects on cognitive function in young and older adults [3,5,6]. However, recent meta-analyses have reported that cognitive training has a small or medium effect size [7]. Therefore, researchers are interested in enhancing the benefits of cognitive training.

A recent study indicated that brain activity during CT would be a key factor in enhancing the beneficial effects of CT [8]. Previous CT studies have reported that brain activity during training tasks at baseline was positively associated with cognitive benefits from CT [4,9]. For example, healthy young adults with greater brain activation at the dorsolateral prefrontal cortex (DLPFC) during brain training games showed more beneficial effects from CT [4]. In addition, healthy older adults showed that functional connectivity in the working-memory network (the right DLPFC as a seed region) during an easy working-memory task (1-back working-memory task) were negatively associated with cognitive benefits from CT [10]. On the other hand, the older adults also showed a positive correlation between the functional connectivity in the working-memory network during a difficult working-memory task (3-back working memory) and cognitive benefits from CT [10]. These findings suggested that participants would receive more benefits from CT when they maintained higher brain activities during a difficult and complex CT or lower brain activities during an easy CT throughout the training period.

Neurofeedback (NF) training is one way to increase/decrease brain activity. NF is a technique that regulates the self-control of brain activities by measuring them and providing a feedback signal in real time [11]. Previous studies have demonstrated that participants can increase/decrease brain activity after NF training [12,13]. NF training alone also led to improvements in cognitive function [14]. In addition, previous studies have suggested that a combination of different training programs has beneficial effects on cognitive functions compared to a single training program [12]. Therefore, CT with NF (CT-NF) would be expected to have more positive effects on cognitive functions because NF would maintain participants’ higher/lower brain activities during CT.

In a previous CT-NF study using NIRS, participants were asked to decrease brain activity in the prefrontal cortex during working-memory training [13]. The study using CT-NF showed significant improvements in working memory and switching performance compared to CT with sham-NF. This finding suggested that CT-NF would have more beneficial effects on cognitive function than CT alone among healthy adults.

A previous study indicated that a CT-NF using NIRS would have a superior benefit on cognitive function compared to CT alone [13]. However, some issues remain unresolved. First, NF has two effective strategies to manipulate brain activities (down-regulation and upregulation) [15]. The prior study using NIRS signal used only downregulate strategy during CT-NF [13]. It is unknown whether the upregulation strategy during CT-NF would have beneficial effects on cognitive function. Second, previous studies performed CT-NF in the laboratory [13]. The participants came to the experimental room to receive CT-NF. Considering moving costs and accessibility, it is important to provide a CT-NF at home. Third, previous studies did not investigate whether CT-NF led to improvements in a wide range of cognitive functions. Previous CT-NF studies measured mainly executive function and working memory [12,13]. However, previous studies have reported that CT leads to improvements in several cognitive functions [6]. In addition, NF has positive effects on a wide range of cognitive functions [14]. Therefore, there is a possibility that CT-NF would enhance a wide range of cognitive functions in healthy young adults.

**Figure 1:**
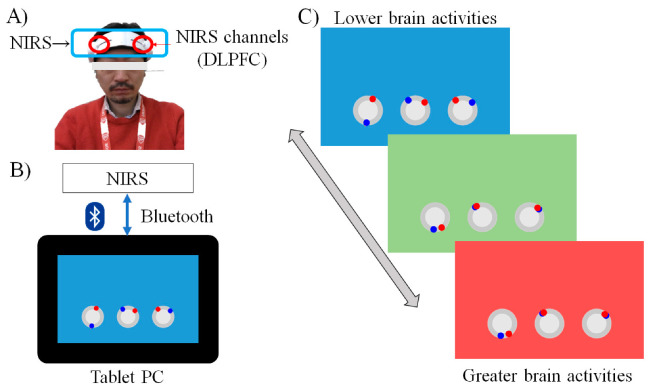
Cognitive training with neurofeedback system. (**A**) The 2 channel NIRS devise set in the bilateral DLPFC. (**B**) The training device contained a tablet PC (MediaPad M3 lite, Huawei, Japan) and a portable NIRS device (HOT-1000, NeU, Japan). A tablet PC was used to perform the training and stored training performance. (**C**) The background color changed in real time from blue to red depending on the neurofeedback signals.

To clarify the abovementioned issues, we conducted a double-blind, randomized controlled trial (RCT) among young adults in three groups: an NF-CT, a CT, and an active control group (ACT). To investigate the first issue, we used an upregulated strategy during CT-NF. A previous study reported that greater brain activity in the DLPFC at baseline was positively associated with greater benefits from CT [4]. Therefore, participants were asked to increase their brain activity in the DLPFC during CT. To resolve this issue, we developed a new portable CT-NF system (Figure 1). The training tasks were video gaming. The system simply used a 2ch NIRS and a tablet. Participants can perform CT-NF at home. To check the third issue, we measured a wide range of cognitive functions, such as executive function, working memory capacity, episodic memory, and processing speed.

Based on the previous results, we created the following three hypotheses. First, the NF-CT would increase the brain activities in the bilateral DLPFC compared to the CT and ACT groups because the NF-CT group is instructed to use the upregulation strategy during training tasks. Second, CT-NF shows the superior benefits of cognitive functions. Third, we expected a significant positive correlation between the increase in brain activity and improvement in cognitive function.

## 2. Materials and Methods

### 2.1. Randomized Controlled Trial Design and Setting of This Trial

This RCT was conducted from November 2017 to May 2018 in Sendai, Japan. The study protocol was approved by the Ethics Committee of Tohoku University Graduate School of Medicine. This RCT was registered at the University Hospital Medical Information Network Clinical Trial Registry (UMIN000034594).

**Figure 2:**
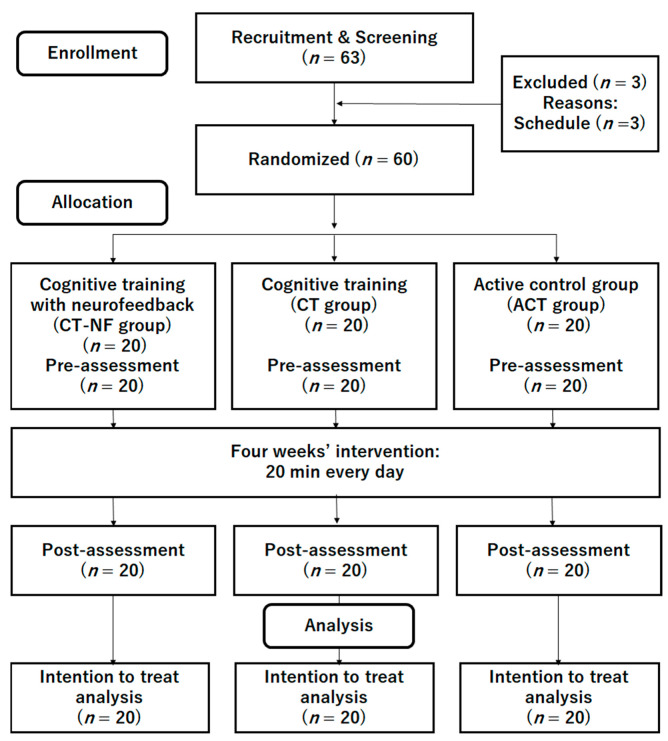
CONSORT diagram.

We conducted a double-blinded RCT with an active control group. All participants and testers were blinded to the study hypotheses and group membership of participants. The Consolidated Standards of Reporting Trials (CONSORT) statement (http://www.consort-statement.org/home/ (accessed on 11 November 2021), see Supplementary Table S1) was used to report the study structure. The RCT design is shown in Figure 2.

### 2.2. Participants

We recruited undergraduate and graduate students using online advertisements at the university. The inclusion and exclusion criteria were written on flyers. A total of 63 interested participants contacted the research group via e-mail (Figure 2) and participated in an orientation meeting. Three participants were excluded because of their schedules. During the meeting, one researcher (R.N.) explained the study details, and all participants provided informed consent. The researcher then checked whether the interested participants were eligible to participate in the study. No patients were excluded at this stage. Then, 60 participants were randomly assigned to either the CT-NF, CT, or ACT groups. Table 1 presents the baseline characteristics of all participants (*n* = 60; 30 males, 30 females; average age = 21.43 years (*SD* = 1.14)). There was no significant difference in the baseline data among the three groups.

### 2.3. Inclusion and Exclusion Criteria

Based on our previous study [6], we used the following inclusion criteria: (1) right-handed; (2) native Japanese speakers; (3) 20–30 years of age; (4) not concerned about their memory functions and not using medications known to interfere with cognitive functions (including benzodiazepines, antidepressants, or other central nervous agents); (5) no history of diseases known to affect the central nervous system, including thyroid disease, multiple sclerosis, Parkinson’s disease, stroke, diabetes, and severe hypertension (systolic blood pressure > 180 mmHg, diastolic blood pressure > 110 mmHg); and (6) non-gamers who reported playing video games less than one hour a week over the previous two years. Participants who had participated in other cognition-related intervention studies were also excluded.

### 2.4. Sample Size

We calculated the sample size using the G Power version 3.1. The sample size was calculated based on the working-memory performance task, which was the primary outcome measure in this study. A previous CT-NF study reported a large effect size (*d* = 0.79) on working memory and executive function performance [13]. Based on this evidence, we expected a large effect on working-memory performance. To calculate the sample size, we used an analysis of covariance (ANCOVA) model with working-memory performance at baseline, sex, and age as covariates, α = 0.05, and power = 0.80. We estimated that the sample size was 60 (20 participants in each group).

### 2.5. Randomization

We randomly assigned the participants to the CT-NF, CT, or ACT groups using an online randomization program (http://www.graphpad.com/quickcalcs/index.cfm, accessed on 8 September 2021). We stratified participants according to sex because there were sex differences in cognitive function [16]. We used blocked randomization (block size, 4) with an allocation ratio of 1:1:1.

### 2.6. Overview of the Intervention

Participants were asked to perform CT-NF, CT, or ACT at home for 20 min every day for four weeks. Our previous study used the same protocol [6]. Based on previous studies [4], we used a puzzle game for the ACT group. We also provided the same training device (tablet PC and NIRS device) for all groups (Figure 1A,B). Therefore, participants in both groups had the same training period and a similar training setting. This reduced the effect of new experiences such as performing cognitive tasks on a new device and the effects of monitoring or maintaining the training schedule.

Before the intervention period, participants received instructions on how to use the device and play the training game. All participants played the game using their own devices. The training duration was recorded using the device. Participants were asked to perform the training for approximately 20 min every day for four weeks. At the end of the training period, participants reported their subjective feelings of satisfaction and enjoyment with the training game on a five-point Likert scale: 1 = strongly disagree, 2 = disagree, 3 = neither agree nor disagree, 4 = agree, 5 = strongly agree [17]. We measured cognitive functions before and after the four-week intervention period. The training device was returned on the assessment day after the completion of the intervention period.

### 2.7. CT-NF and CT Groups

**Figure 3:**
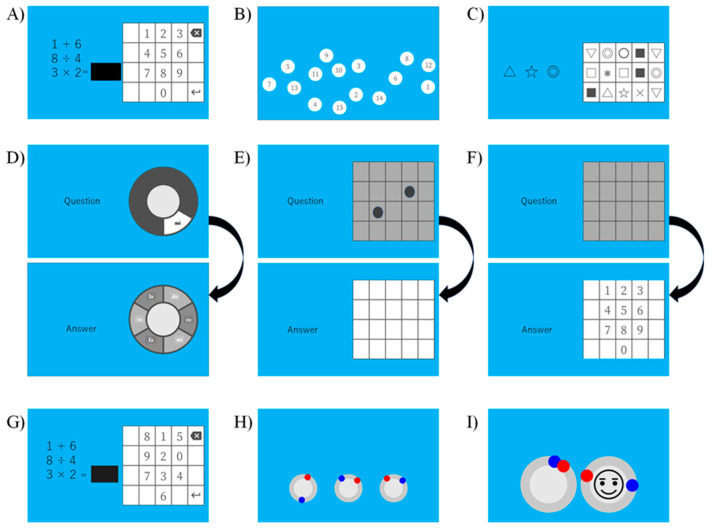
Examples of cognitive training games. (**A**) speed calculation, (**B**) number touch, (**C**) symbol touch, (**D**) name of key span, (**E**) dot matrix span, (**F**) digit number span, (**G**) concentration speed calculation, (**H**) triple circle timing touch, and (**I**) timing touch with a smile.

We developed three types of cognitive training (processing speed, memory span, and attention training) based on previous cognitive training studies [2,3,6,17,18]. Each cognitive training session contained three training games (Figure 3).

The processing speed training included the following: (1) speed calculation (Figure 3A); (2) number touch (Figure 3B); and (3) symbol touch (Figure 3C). In the speed calculation, participants were asked to solve simple mathematical problems as quickly as possible. The problems included four arithmetic operations (addition, subtraction, multiplication, and division). Participants pushed a numeric keypad on the screen to solve them. In the number touch, participants were asked to touch the number from one in sequential order as quickly as possible. In the symbol touch, participants were presented with target symbols and stimuli in a 5 (row) × 3 (columns) matrix. They were asked to touch the target symbols as quickly as possible.

The memory span training includes the following: (4) name of key span (Figure 3D); (5) dot matrix span (Figure 3E); and (6) digit number span (Figure 3F). All memory span training begins with the lowest sequence (one item). The sequences of all training increased and decreased in length according to the participants’ performance. In the name of the key span, a sound of seven pitches on a diatonic scale and the name of the key (Do, Re, Mi, Fa, Sol, La, Si) were presented at the same time. Participants were asked to memorize the presented sound. After the final sound was presented, the participants answered the sequences of the presented sounds using a touch screen. In the dot matrix span, a black dot was presented one at a time in a 5 × 5 matrix. Participants were asked to memorize the location of the dots. After the final dot of the sequence appeared, the participants touched the matrix in the presentation order of the dot. In the digit number span, participants listened to the sequence of a digit number. They were asked to memorize the digit numbers in their presentation order. After the last digit number of each sequence appeared, participants touched the number on the screen in the presented order of digit numbers.

The attention training includes the following: (7) concentration speed calculation (Figure 3G); (8) triple circle timing touch (Figure 3H); and (9) timing touch with a smile (Figure 3I). The concentration speed calculation was almost the same as that of the speed calculation. However, the positions of numbers in a numeric keypad change every problem. Therefore, participants touched the numeric keypad with attention. Next, three circles were presented on the screen in the triple circle timing touch. The red and blue bots moved around the circle at different speeds. The participants were asked to touch the bot when each dot overlapped. In the timing touch with a smile, two circles are presented. The red and blue dots moved around the circle. The participants were asked to touch the dot when each overlapped. In addition, participants were asked to touch a smile mark when the mark appeared.

### 2.8. Active Control Training Group

We used Tetris as the ACT game based on previous studies [3,6]. Tetris is a popular block-puzzle game where blocks drop from the top of the screen, which participants can rotate and move, fitting them together to make a complete line. If the line is completed with no gaps, the participants acquire points. In Tetris, high game scores are acquired by forming complete lines. The active control group was designed to control the use of a new device, play a video game, and maintain an intervention schedule.

### 2.9. Training Device

The training device contained a tablet PC (MediaPad M3 lite, Huawei, Japan) and a portable NIRS device (HOT-1000, NeU, Japan). A tablet PC was used to perform the training and stored training performance. The portable NIRS was used to measure prefrontal brain activities (please see the details of the HOT-1000 device in previous studies). HOT-1000 is a 2ch NIRS with a single wavelength of 810 nm and measures the concentration change in total hemoglobin (total Hb).

Participants were asked to set the two dual source-detector (SD) optodes at the left and right dorsolateral prefrontal cortex (DLPFC: BA46) (Figure 1A), with about 3 cm detector 3 cm left or right from the Fpz position in the 10–20 system [19].

Before the intervention, all participants were introduced to the 10–20 system. The NIRS devise had a marker at the center. Participants were required to set the market on the Fpz position. A researcher (R.N.) explained in detail until they were able to set the NIRS device correctly.

### 2.10. NF Procedure

We used a 30 s rest period and a 60 s task period. During the rest period, the participants were asked to count their breathing while taking deep breathing to control for potentially confounding mental processes [20].

During the task period, the participants were asked to perform CT or ACT. NF signals during the task period were calculated by subtracting the average of total Hb during the rest period from the average total Hb in the last 5 s. The range of the NF signal ranged from 0 to 0.2. The NF signals below 0 were converted to 0. The NF signal was converted to 0.2.

For the CT-NF group, the NF signals were displayed as a background color during the task. The background color changed in real time from blue to red depending on the NF signals (Figure 1C). Additionally, the CT-NF group received a brain activation score at the end of each training session. An average of the NF signals (0–0.2) was converted to the brain activation score (0–100). On the other hand, for the CT alone and ACT groups, the background color did not change during the task period (no feedback).

### 2.11. Cognitive Functions

We investigated the performance of processing speed, attention, inhibition, short-term memory, working memory, and episodic memory. It took approximately 1.5 h to complete all cognitive tests.

To briefly check the participants’ reading ability and IQ, we used the JART [21], a Japanese version of the National Adult Reading Test (NART), consisting of 25 Kanji (Chinese characters) compound words. The reading stimuli were printed randomly for reading. Participants were asked to write the pronunciation of each kanji compound word.

To assess processing speed performance, we used digit symbol coding (Cd) from the WAIS-III [22]. The following descriptions of Cd were reproduced from our earlier report [23]. “For Cd, the participants were shown a series of symbols that were paired with numbers. Using a key within a 120 s time limit, participants drew each symbol under its corresponding number. The primary measure of this test was the number of correct answers”.

To measure inhibition performance, we used a paper-pencil version Stroop task (ST) [24]: “In the ST, in the leftmost of six columns, a word naming a color was printed in another color (e.g., “red” was printed in blue letters), and the other five columns contained word naming colors. Participants were required to check the column containing the word naming the color of the word in the leftmost column. The primary measure for this task was the number of correct items” [23].

To measure attention performance, we conducted a digit cancellation task (D-CAT). The following descriptions of the D-CAT are reproduced from our earlier report [6]: “The test sheet consists of 12 rows of 50 digits. Each row contains five sets of numbers 0–9 arranged in random order. Thus, anyone digit would appear five times in each row with randomly determined neighbors. The D-CAT consists of three sheets. Participants were instructed to search for the target number that had been specified to them and to delete each one with a slash mark as quickly and as accurately as possible for 1 min until the experimenter sent a stop signal. The primary measure of this test was the number of hits”.

To measure short-term memory and working memory capacity, we used the digit span forward (DS-F) and digit span backward (DS-B) tasks. DS-F and DS-B are subtests of the WAIS-III [22]. The following descriptions of the DS-F and DS-B are reproduced from our earlier report [23]: “For the DS-F, participants repeated numbers in the same order as they were read aloud by the examiner. For the DS-B, participants repeated numbers in the reverse order of that presented aloud by the examiner. In both tasks, the examiner read a series of number sequences that the participant was required to repeat in either forward or reverse order”. The primary measure of this test was digit number length. The maximum digit number length in DS-F was 9, and that in DS-B was 8.

To measure episodic memory, we used the logical memory (LM) subtest of the WMS-R [25]: “which consists of two short-paragraph-length stories (Story A and Story B). For the LM activity, participants were required to memorize one of the two stories. The stories were scored in terms of the number of story units recalled, as specified in the WMS-R scoring protocol. We used either Story A or Story B. The primary measure for this task was the number of correct story units recalled” [23]. We checked the performance of the immediate recall and delayed recall memory.

To measure visuospatial performance, we used a paper-pencil version’s mental rotation (MR) test [26]. “The MR test uses three-dimensional cubical figures and has 24 items. Each item comprises a row of five drawings, with a target figure in the leftmost position followed by four response-choice figures. The participants were asked to find the two choice figures that were the rotated reproductions of the target figure” [27].

### 2.12. Data Analyses

All analyses were conducted using the R software (R ver. 4.01). All participants were included on the basis of the intention-to-treat principle. We imputed missing values (m (the number of multiple imputations) = 20) using all variables of cognitive function performance, age, and sex with “mice” in the mice package [28]. We used predictive mean matching because using this method in multiple imputations can work even if the sample size is small [29].

To check the baseline differences in demographic measures and cognitive function among the groups, we used a permutation analysis of variance with the “aovp” function in the lmPerm package (http://cran.r-project.org/web/packages/lmPerm/index.html, accessed on 8 September 2021).

For cognitive function analysis, we calculated the change scores in cognitive functions and emotional states (post-intervention score minus pre-intervention score). To investigate whether CT-NF improved cognitive functions compared to other training groups, we analyzed the all-change scores in cognitive functions using a permutation ANCOVA. In the analyses, we used the pre-score for the dependent variable, age, and sex as covariates. Permutation tests were performed because they were suitable for small sample analyses and were distributed freely. Previous studies used a similar method in RCTs [6,17].

For NIRS data analysis, we used the following preprocessing [4]. First, the NIRS signals were detrended and low-pass filtered (cut-off at 0.1 Hz). Second, we calculated the neural signals using the dual source-detector regression to regress out systemic and motion-related noises [30]. Then, the NIRS signals during training periods were baseline-corrected by subtracting the average NIRS signals of the preceding rest periods. Finally, we calculated the change in NIRS signals (average of the baseline-corrected NIRS signals during the last 3 days (post-NIRS signals) minus that during the first 3 days (pre-NIRS signals)) at the left and right DLPFC in each subject. To investigate whether CT-NF improved brain activity during training compared to other training groups, we separately performed a permutation ANCOVA for the change in NIRS signals at the left and right DLPFC. In these analyses, we adjusted for the effect of pre-NIRS signals, age, and sex.

In these analyses, *p* < 0.05 was considered significant for multiple comparison methods using false discovery rate (FDR) correction methods (Benjamini and Hochberg, 2000).

In addition, we investigated the association between the changes in cognitive functions and changes in brain activities and performed a permutation multiple regression using the “lmp” function in the lmperm package in each group. In the additional analysis, we used the left or right change NIRS signals as the dependent values, the change score in cognitive function as the independent value, and the pre-score in the dependent variable at baseline, age, and sex were used as covariates.

## 3. Results

### 3.1. Training Data

First, we checked the training adherence. There was no significant difference in training days among the groups (CT-NF (*mean* = 25.43, *SD* = 0.84), CT (*mean* = 25.11, *SD* = 0.98), and ACT (*mean* = 24.55, *SD* = 1.06)). In addition, we evaluated changes in training task performance and participants’ satisfaction and enjoyment after the intervention using a 5-point scale. There was no significant difference in the average satisfaction scores among the CT-NF (*mean* = 3.84, *SD* = 0.52), CT (*mean* = 3.81, *SD* = 0.45), and ACT (*mean* = 3.88, *SD* = 0.57) groups and enjoyment scores among the CT-NF (*mean* = 3.99, *SD* = 0.37), CT (*mean* = 3.95, *SD* = 0.43), and ACT (*mean* = 3.98, *SD* = 0.32) groups.

### 3.2. Cognitive Function Data

We investigated the effects of CT-NF on cognitive function using a permutation ANCOVA for change scores (Table 2). Significant group differences were found in episodic memory (*F* (2, 54) = 8.58, *η*^2^ = 0.14, *adjusted p* = 0.002), working memory (*F* (2, 54) = 10.28, *η*^2^ = 0.08, *adjusted p* < 0.001), processing speed (*F* (2, 54) = 5.43, *η*^2^ = 0.12, *adjusted p* = 0.023), attention (*F* (2, 54) = 7.98, *η*^2^ = 0.18, *adjusted p* = 0.004), and mental rotation (*F* (2, 54) = 10.89, *η*^2^ = 0.18, *adjusted p* < 0.001). Post-hoc analysis using Bonferroni method revealed that CT-NF showed greater improvements in LM delay, DS-B, and D-CAT compared to CT and ACT. In addition, the CT-NF and CT groups showed significant improvements in Cd compared to the ACT group. Finally, the ACT group showed greater improvement in MR than the CT-NE and CT groups.

### 3.3. NIRS Data

We analyzed brain activity during the training task using a permutation ANCOVA for change values. Significant group differences were found in the right DLPFC (*F* (2, 54) = 4.21, *η*^2^ = 0.05, *adjusted p* = 0.054) and the left DLPFC (*F* (2, 54) = 5.11, *η*^2^ = 0.08, *adjusted p* = 0.002). The following analysis using Bonferroni method revealed that the CT-NF group showed greater brain activity than the other groups in the left DLPFC (CT-NF (*mean* = 0.17 (*SD* = 0.06)) > CT (*mean* = 0.03 (*SD* = 0.07)) and ACT (*mean* = −0.05 (*SD* = 0.11))) and the right DLPFC (CT-NF (*mean* = 0.14 (*SD* = 0.05)) > CT (*mean* = −0.06 (*SD* = 0.12)) and ACT (*mean* = −0.03 (*SD* = 0.08))).

### 3.4. Correlation between the Changes of Cognitive Function and the Changes in Brain Activities

We analyzed the correlation between the change in NIRS signals and the changes in cognitive functions. We found that the CT-NF group showed significant positive correlations between the increase in left DLPFC activity and improvement in episodic memory (*standardized β* = 0.29, *t* = 2.86, *p* = 0.011), working memory (*standardized β* = 0.33, *t* = 3.16, *p* = 0.006), and attention performance (*standardized β* = 0.26, *t* = 2.66, *p* = 0.017). We did not find any other significant correlations between the changes in cognitive functions and the change of brain activities.

## 4. Discussion

We investigated whether CT-NF using the upregulation would have more beneficial effects on cognitive functions compared with CT alone and ACT. We found three main findings in this study. First, after 4 weeks of training, CT-NF increased brain activity in the left and right DLPF during the training task. Second, the CT-NF groups showed superior beneficial effects on episodic memory, working memory, and attention compared to the CT alone and ACT groups. Finally, we found that only the CT-NF group showed an increase in brain activity that was associated with improvements in cognitive function. The key findings are discussed in separate sections.

The first main finding was that the CT-NF changed brain activity in the bilateral DLPFC during CT. This result is consistent with previous NF-CT-FC studies [12,13]. A previous study using CT-NF used down-regulation instruction for NF [13]. In Hosseini’s study, participants were asked to decrease their brain activity during working-memory training. Participants using the NF with down-regulation showed a significant decrease in brain activity during CT in the prefrontal cortex. However, this study is the first to demonstrate that brain activity increases during CT. Based on the results from the previous study and the current study [13], CT-NF could upregulate and downregulate brain activity during CT.

The second main finding is that CT-NF has beneficial effects on episodic memory, working memory, and attention compared to other training groups. These results support previous findings that reported improvements in cognitive function after CT-NF [13,31,32]. This study is the first to demonstrate improvements in episodic memory and attention after CT-NF. The improvement in working memory is consistent with previous results using CT-NF [13]. In addition, previous studies using working-memory training with NF [13] improved the working-memory performance measured by N-back performance. However, this study showed significant improvements in the working-memory span measured by DS-backward. Taken together, CT-NF may have beneficial effects on a wide range of cognitive functions in young adults.

The third main finding was that there were significant positive correlations between improvements in brain activity and improvements in cognitive functions. These results are consistent with previous findings [13,31]. Previous CT-NF studies using down-regulation instruction have reported that reductions in brain activity were associated with N-back performance after the intervention period [13]. However, this study is the first to demonstrate that an increase in brain activity in the left DLPFC is associated with improvement in episodic memory, working memory, and attention performance.

It is important to consider a possible mechanism that would explain the superior benefits of CT-NF on these cognitive functions compared to CT alone. CT-NF and CT alone used the same training games, such as processing speed, memory span, and attention training. However, the CT-NF asked participants to increase brain activity in the bilateral DLPFC during CT. Previous neuroimaging studies have reported that the DLPFC plays a key role in episodic memory [33], working memory [34], and attention [35]. In addition, previous studies indicated that participants received more benefit from cognitive training when the DLPFC was activated during the training task [4]. Therefore, greater brain activity in the DLPFC during CT is important to enhance episodic memory, working memory, and attention performance.

This study had several limitations. First, the participants were healthy young adults. Previous studies have reported that CT and NF have positive effects on aging and clinical populations [36,37,38,39]. It is important to investigate whether CT-NF leads to improvements in cognitive functions in aging and clinical populations. Second, this study only measured brain activity in the bilateral DLPFC. Previous studies using NF have suggested that NF regulates activities in other brain regions such as the hippocampus and amygdala. In future studies, it would be necessary to investigate whether CT-NF for other brain regions would improve cognitive function. Third, this study did not measure cognitive function at the follow-up. It is still unclear whether the positive effect of CT-NF is maintained for a long time. It is important to investigate the long-term benefits of CT-NF on cognitive function.

## 5. Conclusions

In conclusion, we developed a new CT-NF with a portable system and conducted an RCT to investigate the effects of CT-NF on cognitive functions compared to CT alone and ACT. Our results showed that CT-NF led to increased DLPFC activity after 4 weeks of training. In addition, the CT-NF had superior benefits of improvements in episodic memory, working memory, and attention compared to other training groups. Finally, the increase in left DLPFC activity was positively associated with improvements in all cognitive functions. These results indicate that CT-NF has beneficial effects on cognitive functions and that the activities during training tasks play an important role in the enhancement of cognitive performance.

## Figures and Tables

**Table 1:** Mean of demographic information and cognitive function of all groups at baseline.

	CT-NF	CT	ACT	Effect Size	*p*-Value
Demographic information					
Age	21.25	21.50	21.55	0.01	0.60
	(1.12)	(1.15)	(1.19)		
JART ^(1)^	20.30	20.65	20.45	0.01	0.66
	(1.89)	(1.60)	(1.76)		
Processing speed					
Cd ^(2)^	98.90	98.75	98.45	0.00	0.96
	(12.90)	(12.56)	(8.61)		
Executive functions (inhibition)					
ST ^(2)^	60.45	61.25	65.45	0.02	0.40
	(9.83)	(7.03)	(7.75)		
Attention					
D-CAT ^(2)^	65.20	65.35	64.20	0.00	0.94
	(9.73)	(10.97)	(10.23)		
Short-term memory					
DS-F ^(3)^	7.50	7.20	7.60	0.02	0.60
	(1.24)	(1.01)	(1.19)		
Working memory					
DS-B ^(3)^	6.00	6.10	6.05	0.00	0.88
	(1.45)	(1.41)	(1.61)		
Episodic memory					
LM immediate ^(1)^	16.35	16.35	15.70	0.01	1.00
	(3.47)	(4.00)	(3.06)		
LM delay ^(1)^	15.70	15.90	15.05	0.01	0.82
	(3.06)	(3.73)	(2.98)		
Visuospatial performance					
MR ^(2)^	30.85	30.60	30.75	0.00	1.00
	(6.29)	(7.31)	(6.84)		

Note: CT-NF = cognitive training with neurofeedback group, CT = cognitive training alone group, ACT = active control group, standard deviation (*SD*) in parentheses. ^(1)^ = unit of the test is score, ^(2)^ = unit of the test is correct number, ^(3)^ = unit of the test is digit number, JART = Japanese version of the National Adult Reading Test, Cd = digit symbol coding, ST = Stroop task, D-CAT = digit cancellation task, DS-F = digit span forward, DS-B = digit span backward, LM = logical memory, MR = mental rotation.

**Table 2:** Mean of change scores of cognitive functions in all groups.

				ANCOVA			Post-Hoc Analysis for Group Differences		
	CT-NF	CT	ACT	Effect Size	*p*-Value	*Adjusted* *p*-Value	*Adjusted* *p*-Value (CT-NF vs. CT)	*Adjusted* *p*-Value (CT-NF vs. ACT)	*Adjusted* *p*-Value (CT vs. ACT)
Processing speed									
Cd	16.50	15.15	5.95	0.12	0.015	0.023	1	0.010	0.026
	(13.34)	(10.56)	(15.25)						
Executive functions (inhibition)									
ST ^(1)^	3.90	3.05	0.75	0.03	0.316	0.361	1	1	0.568
	(4.45)	(9.57)	(8.88)						
Attention									
D-CAT ^(1)^	12.00	3.15	5.10	0.18	0.002	0.004	0.000	0.052	1
	(9.77)	(7.64)	(8.41)						
Short-term memory									
DS-F ^(2)^	−0.10	0.50	−0.10	0.01	0.750	0.750	1	1	1
	(1.45)	(1.64)	(1.41)						
Working memory									
DS-B ^(2)^	1.55	0.40	0.45	0.08	0.000	0.000	0.000	0.001	1
	(1.61)	(1.35)	(2.04)						
Episodic memory									
LM immediate ^(3)^	1.30	−0.25	0.60	0.05	0.095	0.127	0.463	0.667	1
	(3.34)	(3.40)	(2.74)						
LM delay ^(3)^	2.45	0.30	0.25	0.14	0.001	0.002	0.010	0.000	1
	(2.84)	(3.08)	(2.77)						
Visuospatial performance									
MR ^(1)^	3.70	2.85	10.65	0.18	0.000	0.000	1	0.003	0.000
	(9.40)	(5.79)	(8.06)						

Note: CT-NF = cognitive training with neurofeedback group, CT = cognitive training alone group, ACT = active control group, Standard deviation (*SD*) in parentheses. ^(1)^ = unit of the test is correct number, ^(2)^ = unit of the test is digit number, ^(3)^ = unit of the test is score. Cd = digit symbol coding, ST = Stroop task, D-CAT = digit cancellation task, DS-F = digit span forward, DS-B = digit span backward, LM = logical memory, MR = mental rotation.

## Data Availability

The data sets used and analyzed in the current study are available from the corresponding author upon reasonable request.

## Supplementary Materials

The following are available online at https://www.mdpi.com/article/10.3390/brainsci12010005/s1, Table S1: Details of CONSORT checklist of this study.

## References

[1] Takeuchi H., Taki Y., Nouchi R., Hashizume H., Sekiguchi A., Kotozaki Y., Nakagawa S., Miyauchi C.M., Sassa Y., Kawashima R. (2013). Effects of working memory training on functional connectivity and cerebral blood flow during rest. Cortex.

[2] Nouchi R., Saito T., Nouchi H., Kawashima R. (2016). Small Acute Benefits of 4 Weeks Processing Speed Training Games on Processing Speed and Inhibition Performance and Depressive Mood in the Healthy Elderly People: Evidence from a Randomized Control Trial. Front. Aging Neurosci..

[3] Nouchi R., Taki Y., Takeuchi H., Hashizume H., Akitsuki Y., Shigemune Y., Sekiguchi A., Kotozaki Y., Tsukiura T., Yomogida Y. (2012). Brain Training Game Improves Executive Functions and Processing Speed in the Elderly: A Randomized Controlled Trial. PLoS ONE.

[4] Nouchi R., Kawata N.Y.D.S., Saito T., Himmelmeier R.M., Nakamura R., Nouchi H., Kawashima R. (2020). Dorsolateral Prefrontal Cortex Activity during a Brain Training Game Predicts Cognitive Improvements after Four Weeks’ Brain Training Game Intervention: Evidence from a Randomized Controlled Trial. Brain Sci..

[5] Brehmer Y., Westerberg H., Bäckman L. (2012). Working-memory training in younger and older adults: Training gains, transfer, and maintenance. Front. Hum. Neurosci..

[6] Nouchi R., Taki Y., Takeuchi H., Hashizume H., Nozawa T., Kambara T., Sekiguchi A., Miyauchi C.M., Kotozaki Y., Nouchi H. (2013). Brain Training Game Boosts Executive Functions, Working Memory and Processing Speed in the Young Adults: A Randomized Controlled Trial. PLoS ONE.

[7] Soveri A., Antfolk J., Karlsson L., Salo B., Laine M. (2017). Working memory training revisited: A multi-level meta-analysis of n-back training studies. Psychon. Bull. Rev..

[8] Baykara E., Könen T., Unger K., Karbach J. (2021). MRI Predictors of Cognitive Training Outcomes. J. Cogn. Enhanc..

[9] Vermeij A., Kessels R.P.C., Heskamp L., Simons E.M.F., Dautzenberg P.L.J., Claassen J.A.H.R. (2017). Prefrontal activation may predict working-memory training gain in normal aging and mild cognitive impairment. Brain Imaging Behav..

[10] Heinzel S., Lorenz R.C., Brockhaus W.-R., Wüstenberg T., Kathmann N., Heinz A., Rapp M.A. (2014). Working Memory Load-Dependent Brain Response Predicts Behavioral Training Gains in Older Adults. J. Neurosci..

[11] Rogala J., Jurewicz K., Paluch K., Kublik E., Cetnarski R., Wróbel A. (2016). The Do’s and Don’ts of Neurofeedback Training: A Review of the Controlled Studies Using Healthy Adults. Front. Hum. Neurosci..

[12] Gordon S., Todder D., Deutsch I., Garbi D., Alkobi O., Shriki O., Shkedy-Rabani A., Shahar N., Meiran N. (2019). Effects of neurofeedback and working memory-combined training on executive functions in healthy young adults. Psychol. Res..

[13] Hosseini S.H., Pritchard-Berman M., Sosa N., Ceja A., Kesler S.R. (2016). Task-based neurofeedback training: A novel approach toward training executive functions. NeuroImage.

[14] Da Silva J.C., De Souza M.L. (2021). Neurofeedback training for cognitive performance improvement in healthy subjects: A systematic review. Psychol. Neurosci..

[15] Ruiz S., Buyukturkoglu K., Rana M., Birbaumer N., Sitaram R. (2014). Real-time fMRI brain computer interfaces: Self-regulation of single brain regions to networks. Biol. Psychol..

[16] McCarrey A.C., An Y., Kitner-Triolo M.H., Ferrucci L., Resnick S.M. (2016). Sex differences in cognitive trajectories in clinically normal older adults. Psychol. Aging.

[17] Nouchi R., Kobayashi A., Nouchi H., Kawashima R. (2019). Newly Developed TV-Based Cognitive Training Games Improve Car Driving Skills, Cognitive Functions, and Mood in Healthy Older Adults: Evidence from a Randomized Controlled Trial. Front. Aging Neurosci..

[18] Takeuchi H., Magistro D., Kotozaki Y., Motoki K., Nejad K.K., Nouchi R., Jeong H., Sato C., Sessa S., Nagatomi R. (2020). Effects of Simultaneously Performed Dual-Task Training with Aerobic Exercise and Working Memory Training on Cognitive Functions and Neural Systems in the Elderly. Neural Plast..

[19] Nozawa T., Sakaki K., Ikeda S., Jeong H., Yamazaki S., Kawata K.H.D.S., Kawata N.Y.D.S., Sasaki Y., Kulason K., Hirano K. (2019). Prior physical synchrony enhances rapport and inter-brain synchronization during subsequent educational communication. Sci. Rep..

[20] Aranyi G., Pecune F., Charles F., Pelachaud C., Cavazza M. (2016). Affective Interaction with a Virtual Character Through an fNIRS Brain-Computer Interface. Front. Comput. Neurosci..

[21] Matsuoka K., Uno M., Kasai K., Koyama K., Kim Y. (2006). Estimation of premorbid IQ in individuals with Alzheimer’s disease using Japanese ideographic script (Kanji) compound words: Japanese version of National Adult Reading Test. Psychiatry Clin. Neurosci..

[22] Wechsler D. (1997). Wechsler Adult Intelligence Scale.

[23] Nouchi R., Taki Y., Takeuchi H., Hashizume H., Nozawa T., Sekiguchi A., Nouchi H., Kawashima R. (2012). Beneficial effects of reading aloud and solving simple arithmetic calculations (learning therapy) on a wide range of cognitive functions in the healthy elderly: Study protocol for a randomized controlled trial. Trials.

[24] Hakoda Y., Watanabe M. (2004). Manual for New Stroop Test II.

[25] Wechsler D.A. (1987). Wechsler Memory Scale Revised.

[26] Peters M., Laeng B., Latham K., Jackson M., Zaiyouna R., Richardson C. (1995). A Redrawn Vandenberg and Kuse Mental Rotations Test—Different Versions and Factors That Affect Performance. Brain Cogn..

[27] Nouchi R., Hu Q., Saito T., dos Santos Kawata N.Y., Nouchi H., Kawashima R. (2021). Brain Training and Sulforaphane Intake Interventions Separately Improve Cognitive Performance in Healthy Older Adults, Whereas a Combination of These Interventions Does Not Have More Beneficial Effects: Evidence from a Randomized Controlled Trial. Nutrients.

[28] Van Buuren S., Groothuis-Oudshoorn K. (2011). mice: Multivariate Imputation by Chained Equations inR. J. Stat. Softw..

[29] Kleinke K. (2018). Multiple Imputation by Predictive Mean Matching When Sample Size Is Small. Methodol..

[30] Luu S., Chau T. (2008). Decoding subjective preference from single-trial near-infrared spectroscopy signals. J. Neural Eng..

[31] DeBettencourt M.T., Cohen J.D., Lee R.F., Norman K.A., Turk-Browne N.B. (2015). Closed-loop training of attention with real-time brain imaging. Nat. Neurosci..

[32] Parsons B., Faubert J. (2021). Enhancing learning in a perceptual-cognitive training paradigm using EEG-neurofeedback. Sci. Rep..

[33] Blumenfeld R.S., Ranganath C. (2007). Prefrontal Cortex and Long-Term Memory Encoding: An Integrative Review of Findings from Neuropsychology and Neuroimaging. Neuroscientist.

[34] Chai W.J., Hamid A.I.A., Abdullah J.M. (2018). Working Memory from the Psychological and Neurosciences Perspectives: A Review. Front. Psychol..

[35] Squire R.F., Noudoost B., Schafer R.J., Moore T. (2013). Prefrontal Contributions to Visual Selective Attention. Annu. Rev. Neurosci..

[36] Marzbani H., Marateb H.R., Mansourian M. (2016). Methodological Note: Neurofeedback: A Comprehensive Review on System Design, Methodology and Clinical Applications. Basic Clin. Neurosci. J..

[37] Trambaiolli L.R., Cassani R., Mehler D.M.A., Falk T.H. (2021). Neurofeedback and the Aging Brain: A Systematic Review of Training Protocols for Dementia and Mild Cognitive Impairment. Front. Aging Neurosci..

[38] Mewborn C.M., Lindbergh C.A., Miller L.S. (2017). Cognitive Interventions for Cognitively Healthy, Mildly Impaired, and Mixed Samples of Older Adults: A Systematic Review and Meta-Analysis of Randomized-Controlled Trials. Neuropsychol. Rev..

[39] Kohl S.H., Mehler D., Lührs M., Thibault R.T., Konrad K., Sorger B. (2020). The Potential of Functional Near-Infrared Spectroscopy-Based Neurofeedback—A Systematic Review and Recommendations for Best Practice. Front. Neurosci..

